# Dietary Intake of Vegetables and Cooking Oil Was Associated With Drug-Induced Liver Injury During Tuberculosis Treatment: A Preliminary Cohort Study

**DOI:** 10.3389/fnut.2021.652311

**Published:** 2021-05-24

**Authors:** Jinyu Wang, Ke Xiong, Lei Xu, Chao Zhang, Shanliang Zhao, Yufeng Liu, Aiguo Ma

**Affiliations:** ^1^Institute of Nutrition and Health, School of Public Health, Qingdao University, Qingdao, China; ^2^Linyi People's Hospital, Linyi, China; ^3^Qingdao Chest Hospital, Qingdao, China

**Keywords:** diet, drug-induced liver injury, tuberculosis, cooking oil, vegetable, cohort study

## Abstract

**Background and Purpose:** Drug-induced liver injury is challenging during tuberculosis treatment. There is no epidemiological data investigating the relation between dietary intake and the risk of drug-induced liver injury during tuberculosis treatment. The aim of this study is to investigate the association of food and nutrient intake with the incidence of tuberculosis-drug-induced liver injury.

**Methods:** A cohort study was conducted in two city-level tuberculosis-specialized hospitals in Linyi City and Qingdao City, China from January 2011 to December 2013. The dietary intake was assessed by a 3-day 24-h food recall survey and a standard food-frequency questionnaire. The liver functions including aspartate aminotransferase (AST) and alanine aminotransferase (ALT) were monitored throughout the 6-month tuberculosis therapy. Liver injury was defined as ALT or AST higher than two times of the upper limit of normal (ULN). Liver dysfunction was defined as ALT or AST higher than the ULN. The ULN for ALT and AST is 40 U/L. Multivariate logistic regression analyses were performed to determine the dietary factors associated with the incidence of liver injury and liver dysfunction.

**Results:** A total of 605 patients were included in the analysis. During the treatment, 8.1% patients exhibited liver injury and 23.3% patients exhibited liver dysfunction. A lower intake of vegetables was associated with a higher risk of liver injury [OR (95% CI): 3.50 (1.52–8.08), *P* = 0.003) and liver dysfunction [OR (95% CI): 2.37 (1.31–4.29), *P* = 0.004], while a lower intake of cooking oil was associated with a lower risk of liver injury [OR (95% CI): 0.44 (0.20–0.96), *P* = 0.040)] and liver dysfunction [OR (95% CI): 0.51 (0.31–0.85), *P* = 0.009].

**Conclusion:** The current study indicated that the higher risks of tuberculosis-drug-induced liver injury and liver dysfunction were statistically associated with decreased vegetable intake and increased cooking oil intake.

## Introduction

Tuberculosis is a communicable disease caused by *Mycobacterium tuberculosis* and the infection mainly happens in the lung (about 85% of the cases). In 2018, WHO estimated that the world tuberculosis burden was 10.0 million (1). The standard tuberculosis treatment consists of a 2-month intensive phase and a 4-month continuation phase. In the intensive phase, four antibiotics were used, including isoniazid (INH), rifampin (RIF), pyrazinamide (PZA), and ethambutol (EMB); in the continuation phase, INH and RIF were used (2). This long-term and high-dose antibiotic treatment is effective but hepatotoxic.

Liver injury is defined as aspartate aminotransferase (AST) or alanine aminotransferase (ALT) two times higher than its upper limit of normal (ULN) (3). Liver dysfunction was defined as higher than the ULN. The ULN for both AST and ALT was 40 U/L (4). During tuberculosis treatment, 5–30% patients exhibited different levels of liver injury (5–7). Liver injury is usually accompanied by nausea, vomiting, abdominal pain and asthenia, while more severe liver injury can lead to treatment withdraw, reduced treatment efficacy, drug resistance and acute liver failure (8).

Tuberculosis patients are characterized by malnutrition including low body mass index (BMI), deficiency of protein and multiple vitamins (9–12). Malnutrition also plays an important role in drug-induced liver injury (13–15). Two cohort studies reported an association between a low BMI and an increased incidence of liver injury during tuberculosis treatment (14, 16). It is pivotal to understand the associations between dietary factors and tuberculosis-drug-induced liver injury.

However, to our knowledge, no epidemiological study has investigated the association between dietary intake and the risk of tuberculosis-drug-induced liver injury. For other liver diseases, a meta-analysis including 9 cohort studies concluded that a higher vegetable intake was associated with a 39% reduction in the risk of liver cancer (17). Cohort studies suggested that a higher consumption of fruits and vegetables (18) and nuts (19) was associated with a lower non-alcoholic fatty liver disease (NAFLD) risk. Randomized controlled trial indicated that whole grain consumption alleviated NAFLD (20).

The aim of this study is to investigate the associations between dietary food and nutrient intake and the incidence of tuberculosis-drug-induced liver injury by a cohort study.

## Materials and Methods

### Ethics

The study was approved by the Ethic Committee of Medicine of Qingdao Center of Disease Control and Prevention (2009-4). The study was conducted in accordance with the Declaration of Helsinki. All participants provided written informed consents. The trial was registered at the Chinese Clinical Trial Registry (No. ChiCTR-OCC-10000994).

### Study Design and Population

The study was conducted from January 2011 to December 2013 in two hospitals located in Linyi City and Qingdao City, Shandong Province, China. Qingdao is a coastal city, while Linyi is an inland city. The inclusion criteria were: (1) newly diagnosed as pulmonary tuberculosis. The pulmonary tuberculosis was diagnosed by a combination of sputum smear, computed tomography scan and clinical symptoms (e.g., cough, haemoptysis, weight loss, fever and night sweat etc.); (2) ≥18 years old. The exclusion criteria were: (1) pregnant or lactating; (2) combined with other diseases including gastrointestinal, cardiovascular, respiratory diseases, cancer, liver diseases (such as hepatitis B or C, alcohol hepatitis etc.), HIV or mental diseases; (3) taking nutritional supplements in the previous 2 months; (4) drug-resistant tuberculosis; (5) elevated liver indices (AST or ALT higher than 40 U/L).

### Procedure

At entry to the hospital, the weight and height were measured and the demographic information was collected using a standard questionnaire, including sex, age, diabetes, the history of liver diseases, outdoor exercise, education level etc.

Two weeks after starting the treatment, 24-h dietary recalls were conducted by investigators for three consecutive days including two weekdays and one weekend. The participants were asked to recall the food intake and its portion size. The investigators were trained to be familiar with the common local food and used a standard questionnaire to record. The nutrients intake was calculated using the China Food Composition Tables (21). Two months after starting the treatment, a semi-quantitative food frequency questionnaire (FFQ) was conducted to assess the dietary intake in the previous 2 months. The FFQ was modified from a previously validated FFQ for Chinese population (22). The FFQ included 26 items, including white rice, white flour, millet, bran, corn, tofu, beans, soybean milk, egg, chicken, duck, beef, pork, lamb, fish, shrimp, potato, sweet potato, taro, yam, dark vegetable, light vegetable, cooking oil, fruit, tea, dairy products and alcohol. Food consumption frequency was classified as “three times a day,” “twice a day,” “once a day,” “four to five times a week,” “two to three times a week,” “once a week,” “once every 2 weeks,” “once a month,” “once every 2 months,” and “almost never.” The amount of consumed foods was estimated in terms of the Chinese *liang* (equivalent to 50 grams). The amount of consumed tea was estimated in cups. The 26 items were classified into 15 food categories, including refined grains, whole grains, poultry, meat, vegetables, starchy vegetables, fruits, tofu and beans, egg, fish, cooking oil, dairy, shrimp, tea and alcohol.

All participants received the same standard tuberculosis treatment (2). The treatment consists of a 2-month intensive phase using INH, RIF, PZA and EMB, followed by a 4-month continuation phase using INH and RIF. The dosage for INH, RIF, PZA, and EMB were 0.3, 0.45, 0.75, and 1.5 g/d, respectively. The AST, ALT and albumin (ALB) were routinely checked by the hospital staff before the treatment, at 1, 2, and 6 months after starting the treatment. The liver function results were obtained from the hospital. Liver injury was defined as ALT or AST higher than two times of the ULN (3). Liver dysfunction was defined as ALT or AST higher than the ULN. The ULN for ALT and AST is 40 U/L (4).

### Statistical Analysis

The intergroup difference was tested by a Mann–Whitney U-test for non-normal data, a *t*-test for normal data, and a χ^2^-test or a Fisher's exact-test for categorical data. Multivariate logistic analysis was conducted to investigate the dietary factors associated with the incidence of liver injury and liver dysfunction. The highest quantile of the intake of each food was used as the reference group. The age, sex, area, energy intake, BMI, diabetes, education level, and outdoor exercise were adjusted as covariates. The statistical analysis was conducted using SPSS 22.0. *P* < 0.05 was considered statistically significant.

## Results

**Figure 1:**
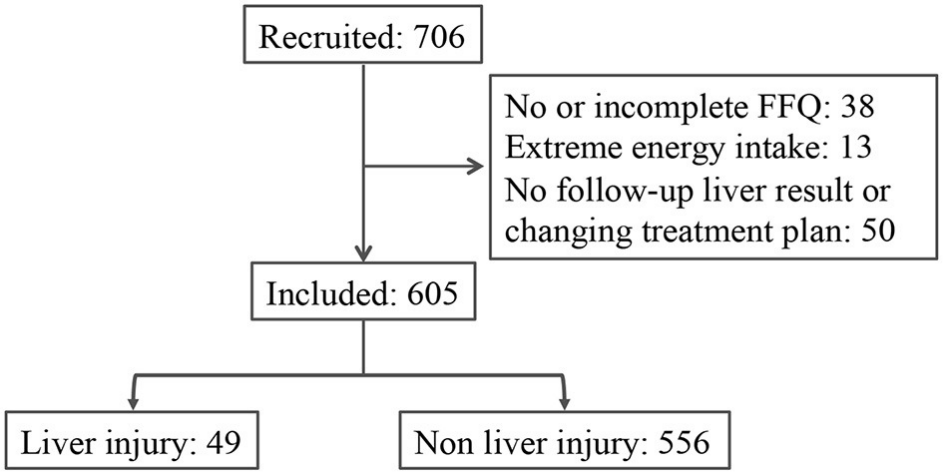
The trial flowchart.

A total of 706 patients were recruited from two hospitals located in Linyi City and Qingdao City, China. Among all the participants, 38 had no or incomplete dietary information (FFQ and 3-day 24-h food recall), 13 reported extreme energy intake, and 50 did not have any follow-up liver function record or changing treatment plan. The median follow-up period was 180 days. During the follow-up, 49 patients exhibited liver injury; 141 patients exhibited liver dysfunction (Figure 1). The characteristics of liver injury and liver dysfunction events were presented in Supplementary Table 1. The median ALT/AST ratios for the liver injury and liver dysfunction group were 1.5 and 1.6, respectively. In comparison, the median ALT/AST ratios for the non-liver-injury and normal liver function group were 1.2 and 1.1, respectively.

The baseline characteristics were compared between the patients with and without liver injury during tuberculosis treatment (Table 1). The patients with liver injury had significantly higher education levels and more Qingdao residents compared to the patients in the non-liver-injury group. The other demographic parameters including age, gender, BMI, smoking, alcohol consumption, marital status, the history of liver diseases, diabetes and outdoor exercise were similar between the liver injury group and the non-liver-injury group. Compared to the patients in the normal liver function group, the patients in the liver dysfunction group had higher education levels, more Qingdao residents and less outdoor exercise.

**Table 1:** The baseline characteristics of the participants classified by liver function.

	**Non-liver-injury group**^**a**^		**Liver injury group**			**Normal liver function group**		**Liver dysfunction group**		
	***n***	**Value**^**b**^	***n***	**Value**	***P***^**c**^	***n***	**Value**	***n***	**Value**	***P***
Age	556	50.0 (35.8)	49	43.0 (30.0)	0.153	464	50.5 (37.0)	141	46.0 (28.5)	0.054
Gender	556		49		0.429	464		141		0.107
Male		402 (72.3%)		38 (77.6%)			330 (71.1%)		110 (78.0%)	
Female		154 (27.7%)		11 (22.4%)			134 (28.9%)		31 (22.0%)	
Area					**0.000**	464		141		**0.000**
Qingdao		179 (32.2%)		31 (63.3%)			129 (27.8%)		81 (57.4%)	
Linyi		377 (67.8%)		18 (36.7%)			335 (72.2%)		60 (42.6%)	
BMI, kg/m^2^	551	20.8 ± 2.9	49	21.4 ± 3.0	0.133	459	20.7 ± 2.8	141	21.1 ± 3.3	0.154
Smoking	556	119 (21.4%)	49	10 (20.4%)	1.000	464	104 (22.4%)	141	25 (17.7%)	0.290
Alcohol consumption	556	88 (15.8%)	49	8 (16.3%)	0.841	464	69 (14.9%)	141	27 (19.1%)	0.237
Marital status	549		49		0.592	457		141		0.424
Single		127 (23.1%)		11 (22.4%)			110 (24.1%)		28 (19.9%)	
Married		402 (73.2%)		38 (77.6%)			330 (72.2%)		110 (78.0%)	
Widowed		17 (3.1%)		0 (0.0%)			15 (3.3%)		2 (1.4%)	
Divorced		3 (0.5%)		0 (0.0%)			2 (0.4%)		1 (0.7%)	
Education completed	550		48		**0.001**	458		140		**0.000**
None		78 (14.2%)		2 (4.2%)			76 (16.6%)		4 (2.9%)	
Primary school		134 (24.4%)		4 (8.3%)			112 (24.5%)		26 (18.6%)	
Class VII-IX		203 (36.9%)		20 (41.7%)			164 (35.8%)		59 (42.1%)	
Class X-XII		92 (16.7%)		12 (25.0%)			73 (15.9%)		31 (22.1%)	
Diploma or higher		43 (7.8%)		10 (20.8%)			33 (7.2%)		20 (14.3%)	
History of liver diseases^d^	550	6 (1.1%)	49	2 (4.1%)	0.134	458	5 (1.1%)	141	3 (2.1%)	0.400
Diabetes	554	87 (15.7%)	49	13 (26.5%)	0.051	462	70 (15.2%)	141	30 (21.3%)	0.087
Outdoor exercise	553		49		0.386	461		141		**0.004**
Almost none		24 (4.3%)		3 (6.1%)			19 (4.1%)		8 (5.7%)	
<2 h per day		181 (32.7%)		20 (40.8%)			139 (30.2%)		62 (44.0%)	
>2 h per day		348 (62.9%)		26 (53.1%)			303 (65.7%)		71 (50.4%)	

a *Liver injury group had an alanine aminotransferase (ALT) or aspartate aminotransferase (AST) level of higher than two times of the upper limit of normal (ULN); Non-liver-injury group had an ALT and AST level of equal or lower than two times of the ULN. Liver dysfunction had an ALT or AST level of higher than the ULN; Normal liver function group had an ALT and AST level of equal or lower than the ULN. The ULN for ALT and AST is 40 U/L*.

b *Non-normal variables were presented as median (IQR); normal variables were presented as mean ± SD; categorical variables were presented as number of patients in a specific category (percentage)*.

c *P was calculated between the non-liver-injury group and liver injury group, and between the normal liver function group and liver dysfunction group. The statistical difference for the non-normal data was tested by a Mann-Whitney U-test; the difference for the normal data was tested by a t-test; the difference for the categorical data was tested by a χ^2^-test*.

d *The intergroup difference for the history of liver diseases was tested by a Fisher's exact-test. P values were labeled in bold if they were less than 0.05*.

The estimated total energy and macronutrients intake were compared between the patients with and without liver injury, and between the patients with and without liver dysfunction (Table 2). The result indicated a similar intake of total energy, carbohydrate and protein, while the intake of fat was significantly higher in the liver injury group compared to the non-liver-injury group (*P* = 0.028), and significantly higher in the liver dysfunction group compared to the normal liver function group (*P* = 0.002).

**Table 2:** The estimated daily macronutrients intake for the participants.

	**Non-liver-injury group^**a**^ (*n* = 556)**	**Liver injury group (*n* = 49)**	***P***^**c**^	**Normal liver function group (*n* = 464)**	**Liver dysfunction group (*n* = 141)**	***P***
Total energy, kcal	1,596.6 (796.1)^b^	1,597.0 (637.9)	0.795	1,578.5 (786.6)	1,625.6 (807.3)	0.218
Carbohydrates, g	263.9 (148.5)	253.2 (99.0)	0.196	265.0 (144.7)	253.6 (137.6)	0.798
Protein, g	65.1 (35.3)	64.3 (36.5)	0.540	64.4 (34.5)	65.8 (40.0)	0.378
Fat, g	23.3 (27.7)	30.3 (38.2)	**0.028**	22.9 (25.3)	27.6 (38.6)	**0.002**

a *Liver injury group had an alanine aminotransferase (ALT) or aspartate aminotransferase (AST) level of higher than two times of the upper limit of normal (ULN); Non-liver-injury group had an ALT and AST level of equal or lower than two times of the ULN. Liver dysfunction had an ALT or AST level of higher than the ULN; Normal liver function group had an ALT and AST level of equal or lower than the ULN. The ULN for ALT and AST is 40 U/L*.

b *Numerical variables are presented as median (IQR)*.

c *P was calculated between the non-liver-injury group and liver injury group, and between the normal liver function group and liver dysfunction group. The statistical difference was tested by a Mann-Whitney U-test. P values were labeled in bold if they were less than 0.05*.

A binary logistic regression was conducted to explore the association between dietary food intake and the risk of liver injury during tuberculosis treatment (Table 3). The model was adjusted for covariates including sex, age, energy intake, area, diabetes, education level, BMI and outdoor exercise. A lower intake of vegetables was associated with a higher risk of liver injury during tuberculosis treatment. Compared to the highest quantile of vegetable intake, the odds ratio (OR) of the middle quantile was 3.50 [95% confidence interval (CI): 1.52–8.08, *P* = 0.003]. However, the lowest quantile of vegetable intake was not associated with the risk of liver injury [OR (95% CI): 1.36 (0.44–4.18), *P* = 0.588]. A lower intake of cooking oil was associated with a lower risk of liver injury during tuberculosis treatment [OR (95% CI): 0.44 (0.20–0.96), *P* = 0.040)]. The intake of refined grains, whole grains, poultry, meat, starchy vegetables, fruits, tofu and beans, egg, fish, dairy, shrimp, and tea was not associated with the risk of liver injury. No alcohol intake was reported in our participants.

**Table 3:** Dietary food intake in relation to the risk of liver injury and liver dysfunction during tuberculosis treatment^a^.

**Quantile of food intake**^**b**^	***n***	**Liver injury OR (95%CI)**	***P***^**c**^	***n***	**Liver dysfunction OR (95%CI)**	***P***
**Refined grain (g/d)**						
<228.6	216	1.90 (0.77–4.70)	0.166	216	0.77 (0.45–1.30)	0.320
228.6–414.3	187	2.02 (0.81–5.02)	0.132	187	1.01 (0.59–1.72)	0.985
≥414.3	185	Reference category		185	Reference category	
**Whole grain (g/d)**						
<6.7	207	0.48 (0.21–1.12)	0.089	207	1.14 (0.67–1.92)	0.635
6.7–57.1	165	0.61 (0.29–1.28)	0.187	165	1.12 (0.68–1.83)	0.661
≥57.1	216	Reference category		216	Reference category	
**Poultry (g/d)**						
<5.0	196	0.78 (0.33–1.84)	0.570	196	1.13 (0.67–1.91)	0.657
5.0–14.3	194	0.92 (0.45–1.91)	0.833	194	0.62 (0.37–1.04)	0.071
≥14.3	198	Reference category		198	Reference category	
**Meat (g/d)**						
<14.3	209	1.13 (0.44-2.87)	0.799	209	1.15 (0.65–2.04)	0.631
14.3–50.0	141	2.16 (0.99–4.74)	0.054	141	1.46 (0.84–2.54)	0.175
≥50.0	138	Reference category		138	Reference category	
**Vegetable (g/d)**						
<78.6	129	1.36 (0.44–4.18)	0.588	129	2.10 (1.11–3.95)	**0.022**
78.6–200.0	113	3.50 (1.52–8.08)	**0.003**	113	2.37 (1.31–4.29)	**0.004**
≥200.0	346	Reference category		346	Reference category	
**Starchy vegetable (g/d)**						
<13.3	182	0.82 (0.35–1.94)	0.654	182	0.76 (0.44–1.31)	0.323
13.3–42.9	188	0.80 (0.38–1.67)	0.553	188	0.72 (0.44–1.17)	0.187
≥42.9	218	Reference category		218	Reference category	
**Fruit (g/d)**						
<14.3	188	0.69 (0.29–1.64)	0.406	188	0.75 (0.43–1.29)	0.293
14.3–71.4	182	0.90 (0.43–1.92)	0.788	182	0.86 (0.52–1.42)	0.558
≥71.4	218	Reference category		218	Reference category	
**Tofu and bean (g/d)**						
<21.4	162	1.00 (0.47–2.14)	0.998	162	0.87 (0.52–1.47)	0.606
21.4–42.9	241	0.75 (0.36–1.56)	0.433	241	0.80 (0.49–1.29)	0.350
≥42.9	185	Reference category		185	Reference category	
**Egg (g/d)**						
<42.9	222	1.00 (0.48–2.11)	0.998	222	1.18 (0.73–1.93)	0.502
42.9–57.1	141	0.69 (0.32–1.52)	0.364	141	0.69 (0.40–1.18)	0.174
≥57.1	225	Reference category		225	Reference category	
**Fish (g/d)**						
<3.3	159	1.20 (0.49–2.93)	0.684	159	1.64 (0.91–2.94)	0.098
3.3–14.3	262	1.12 (0.54–2.32)	0.768	262	1.33 (0.81–2.19)	0.268
≥14.3	167	Reference category		267	Reference category	
**Cooking oil (g/d)**						
<58.3	211	0.44 (0.20–0.96)	**0.040**	211	0.51 (0.31–0.85)	**0.009**
58.3–100.0	167	0.73 (0.35–1.54)	0.411	167	0.63 (0.38–1.05)	0.078
≥100.0	210	Reference category		210	Reference category	
**Dairy (g/d)**						
0	258	1.17 (0.53–2.57)	0.702	258	1.06 (0.64–1.74)	0.833
0.01–64.3	137	1.74 (0.81–3.74)	0.158	137	1.26 (0.74–2.15)	0.392
>64.3	193	Reference category		193	Reference category	
**Shrimp**						
No	385	0.88 (0.42–1.81)	0.719	385	0.94 (0.58–1.54)	0.818
Yes	203	Reference category		203	Reference category	
**Tea**						
No	554	0.82 (0.26–2.59)	0.735	554	0.96 (0.42–2.18)	0.915
Yes	34	Reference category		34	Reference category	

a *Multivariate logistic regression models were used to assess the odds ratios (ORs) and 95% confidence intervals (CIs) for tuberculosis-drug-induced liver injury and liver dysfunction. The model was adjusted by age, sex, area, energy intake, diabetes, education level, BMI and outdoor exercise*.

b *The intake of each food category was divided into three quantiles, while the intake of shrimp and tea was divided into two groups. The highest quantile was used as the reference group*.

c *P was calculated with the use of multivariate logistic regression model for liver injury and liver dysfunction. P values were labeled in bold if they were less than 0.05*.

A similar regression was conducted to explore the association between dietary food intake and the risk of liver dysfunction during tuberculosis treatment (Table 3). A lower intake of vegetables was associated with a higher risk of liver dysfunction during tuberculosis treatment. Compared to the highest quantile of vegetables intake, the OR of the lowest quantile was 2.10 (95% CI: 1.11–3.95, *P* = 0.022) and the OR of the middle quantile was 2.37 (95% CI: 1.31–4.29, *P* = 0.004). The trend was significant (*P*_trend_ = 0.004). A lower intake of cooking oil was associated with a lower risk of liver dysfunction during tuberculosis treatment [OR (95% CI): 0.51 (0.31–0.85), *P* = 0.009)]. The trend was also significant (*P*_trend_ = 0.007). The intake of other foods was not associated with the risk of liver dysfunction.

A binary logistic regression was also conducted to explore the association between the calculated nutrient (e.g., protein, carbohydrate, dietary fiber, fat, vitamins, and minerals) intake and the risk of liver injury or liver dysfunction during tuberculosis treatment (Table 4). The result indicated that the included nutrient intake was not associated with the risk of liver injury or liver dysfunction during tuberculosis treatment.

**Table 4:** Nutrients intake in relation to the risk of liver injury and liver dysfunction during tuberculosis treatment^a^.

**Quantile of nutrient intake**^**b**^	***n***	**Liver injury OR (95%CI)**	***P***^**c**^	***n***	**Liver dysfunction OR (95%CI)**	***P***
**Protein (g/d)**						
<53.70	192	1.60 (0.54–4.72)	0.397	192	1.29 (0.67–2.48)	0.444
53.70–75.90	198	1.25 (0.48–3.28)	0.646	198	1.08 (0.60–1.93)	0.801
≥75.90	198	Reference category		198	Reference category	
**Fat (g/d)**						
<17.00	175	0.61 (0.23–1.66)	0.335	175	1.04 (0.56–1.96)	0.895
17.00–33.40	209	0.71 (0.33–1.54)	0.382	209	0.97 (0.58–1.63)	0.918
≥33.40	204	Reference category		204	Reference category	
**Carbohydrate (g/d)**						
<223.37	195	1.68 (0.50–5.68)	0.402	195	1.42 (0.72–2.79)	0.316
223.37–315.28	195	2.43 (0.85–6.97)	0.098	195	1.39 (0.76–2.54)	0.292
≥315.28	198	Reference category		198	Reference category	
**Dietary fiber (g/d)**						
<8.80	193	0.95 (0.37–2.42)	0.908	193	1.28 (0.71–2.30)	0.416
8.80–15.10	196	0.96 (0.40–2.27)	0.921	196	0.92 (0.53–1.60)	0.768
≥15.10	199	Reference category		199	Reference category	
**Vitamin A (ug RAE/d)**^**d**^						
<207.00	193	0.95 (0.40–2.28)	0.913	193	1.21 (0.69–2.13)	0.515
207.00–390.30	197	0.68 (0.33–1.41)	0.301	197	1.30 (0.80–2.10)	0.294
≥390.30	198	Reference category		198	Reference category	
**Thiamine (mg/d)**						
<0.90	188	0.83 (0.28–2.48)	0.739	188	0.94 (0.49–1.81)	0.846
0.90–1.40	198	0.71 (0.27–1.86)	0.487	198	0.78 (0.44–1.40)	0.407
≥1.40	202	Reference category		202	Reference category	
**Nicotinic acid (mg NE/d)**						
<9.50	193	1.17 (0.43–3.17)	0.756	193	1.19 (0.65–2.19)	0.574
9.50–14.20	197	1.09 (0.45–2.65)	0.843	197	0.74 (0.42–1.30)	0.296
≥14.20	198	Reference category		198	Reference category	
**Riboflavin (mg/d)**						
<0.60	169	1.76 (0.67–4.63)	0.249	169	1.45 (0.78–2.67)	0.237
0.60–0.80	187	1.64 (0.74–3.65)	0.225	187	1.18 (0.70–1.99)	0.539
≥0.80	232	Reference category		232	Reference category	
**Vitamin C (mg/d)**						
<35.50	194	0.79 (0.33–1.88)	0.591	194	1.49 (0.87–2.55)	0.142
35.50–69.40	197	0.98 (0.47–2.03)	0.958	197	1.17 (0.71–1.94)	0.533
≥69.40	197	Reference category		197	Reference category	
**Vitamin E (mg** **α**–**TE/d)**						
<8.80	189	0.86 (0.34–2.13)	0.739	189	1.16 (0.63–2.12)	0.641
8.80–15.20	198	0.36 (0.14–0.93)	**0.034**	198	1.00 (0.57–1.75)	0.998
≥15.20	201	Reference category		201	Reference category	
**K (mg/d)**						
<1,221.00	194	1.21 (0.46–3.18)	0.701	194	1.30 (0.70–2.38)	0.405
1,221.00–1,808.50	197	1.15 (0.48–2.76)	0.760	197	1.04 (0.59–1.82)	0.897
≥1,808.50	197	Reference category		197	Reference category	
**Na (mg/d)**^**e**^						
<239.20	192	0.73 (0.28–1.92)	0.528	192	0.71 (0.39–1.29)	0.257
239.20–598.40	198	0.65 (0.31–1.34)	0.243	198	0.75 (0.47–1.22)	0.246
≥598.40	198	Reference category		198	Reference category	
**Ca (mg/d)**						
<230.00	192	1.25 (0.50–3.10)	0.634	192	1.31 (0.73–2.33)	0.365
230.00–369.50	196	1.28 (0.57–2.87)	0.548	196	1.10 (0.65–1.86)	0.734
≥368.50	200	Reference category		200	Reference category	
**Mg (mg/d)**						
<243.00	193	1.39 (0.49–3.99)	0.539	193	1.49 (0.78–2.85)	0.226
243.00–355.60	195	1.21 (0.46–3.18)	0.702	195	1.45 (0.80–2.61)	0.221
≥355.60	200	Reference category		200	Reference category	
**P (mg/d)**						
<839.90	194	1.93 (0.70–5.34)	0.207	194	1.25 (0.66–2.36)	0.497
839.90–1,173.90	195	0.79 (0.29–2.15)	0.640	195	0.81 (0.45–1.46)	0.477
≥1,173.90	199	Reference category		199	Reference category	
**Fe (mg/d)**						
<15.00	190	0.96 (0.39–2.40)	0.935	190	0.89 (0.49–1.60)	0.693
15.00–23.40	198	1.17 (0.50–2.70)	0.721	198	1.04 (0.61–1.78)	0.889
≥23.40	200	Reference category		200	Reference category	
**Zn (mg/d)**						
<7.70	191	0.56 (0.21–1.54)	0.261	191	1.03 (0.55–1.93)	0.934
7.70–11.00	194	0.50 (0.21–1.21)	0.123	194	0.82 (0.47–1.44)	0.485
≥11.00	203	Reference category		203	Reference category	
**Cu (mg/d)**						
<1.40	192	1.30 (0.44–3.86)	0.641	192	0.95 (0.50–1.82)	0.880
1.40–2.10	184	1.02 (0.39–2.71)	0.964	184	0.96 (0.54–1.72)	0.893
≥2.10	212	Reference category		212	Reference category	
**Se (ug/d)**						
<34.50	191	1.92 (0.74–4.99)	0.180	191	1.04 (0.56–1.92)	0.913
34.50–49.80	196	1.25 (0.54–2.86)	0.600	196	1.12 (0.66–1.88)	0.678
≥49.80	201	Reference category		201	Reference category	
**Mn (mg/d)**						
<4.80	195	1.06 (0.37–3.01)	0.911	195	0.98 (0.53–1.83)	0.951
4.80–7.20	194	1.10 (0.43–2.83)	0.846	194	0.68 (0.38–1.23)	0.204
≥7.20	199	Reference category		199	Reference category	

a *Multivariate logistic regression models were used to assess the odds ratios (ORs) and 95% confidence intervals (CIs) for tuberculosis-drug-induced liver injury and liver dysfunction. The model was adjusted by age, sex, area, energy intake, diabetes, education level, BMI and outdoor exercise*.

b *The intake of each nutrient was divided into three quantiles. The highest quantile was used as the reference group*.

c *P was calculated with the use of multivariate logistic regression model for liver injury and liver dysfunction*.

d *1 ug RAE = 1 ug all-trans-retinol + (1/12) ug all-trans-β-carotene + (1/24) ugprovitamin A carotenoid; 1 mg NE = 1 mg niacin + (1/60) mg tryptophan; 1 mg α-TE = 1 mg α-tocopherol + 0.5 mg β-tocopherol + 0.1 mg γ-tocopherol + 0.02 mg δ-tocopherol + 0.3 mg α-tocotrienol*.

e *The intake of Na as salt was not counted. P values were labeled in bold if they were less than 0.05*.

## Discussions

The current results indicated that a lower vegetable intake and a higher cooking oil intake were associated with an increased risk of liver injury and liver dysfunction during tuberculosis treatment. These findings provided support to recommend high vegetable and low cooking oil intake during tuberculosis treatment.

We first reported here a negative association between vegetable consumption and the incidence of tuberculosis-drug-induced liver injury and liver dysfunction. Similar association has been observed between vegetable consumption and the risk of liver cancer and non-alcoholic liver disease (17, 18). In addition, animal studies indicated that vegetable extract can alleviate xenobiotic-induced hepatic damage (23, 24). Several plant-derived extracts have been shown to relieve tuberculosis-drug-induced liver injury including *Spirulina maxima, Crocus sativus, Ficus religiosa, Mucuna pruriens, Cassia auriculata*, and *Ziziphus oenoplia* (25–30).

Vegetables are rich in phytochemicals (including phenolics, flavonoids, and carotenoids), vitamins (vitamin C, folate, and pro-vitamin A), minerals (potassium, calcium, and magnesium) and fibers (31). Our study did not observe an association between the vitamin, mineral and fiber intake and the risk of liver injury. Phytochemicals may play an important role in alleviating tuberculosis-drug-induced liver injury as shown in the current work. Previous *in-vitro* and animal studies pointed out a protective effect of phytochemicals on liver by increasing hepatic glutathione content, scavenging free radicals, modulating phase II hepatic metabolism, inhibiting the transcription and translocation of nuclear factor-kappa B and reducing pro-inflammatory cytokines (32–34). A 6-month randomized controlled trial indicated that the vegetable and fruit consumption increased the concentration of plasma antioxidants (35). Antioxidant capacity is critical in detoxifying reactive oxygen species and reaction metabolites from tuberculosis drugs (8). Future work should further elucidate the role of common phytochemicals from vegetables on alleviating drug-induced liver injury.

Our study observed a positive association between the cooking oil intake and the risk of liver injury and liver dysfunction during tuberculosis treatment. Vegetable oils, including peanut oil, soybean oil, corn oil and rapeseed oil, are the main cooking oil in China, accounting for 92% dietary oil intake (36). These vegetable oils contain a large amount of unsaturated fatty acids (usually higher than 80% of total fatty acids) with linoleic acid as the major type (37). The consumption of unsaturated fatty acid, especially linoleic acid, increased cytochrome P450 2E1 (CYP2E1) activity, damaged intestinal barrier, and induced liver inflammation (38, 39). CYP2E1 is critical in the hepatotoxicity of tuberculosis drugs which metabolizes isoniazid to reactive oxygen species and diazohydroxide (40). These metabolites are toxic and lead to liver necrosis (40). The activated CYP2E1, increased endotoxemia and liver inflammation by the consumption of unsaturated fatty acids can increase the susceptibility to drug-induced liver injury, and may therefore account for the positive relationship between dietary oil consumption and liver injury during tuberculosis treatment (41, 42).

Our study has several strengths. First, this is the first epidemiological study, which investigates the relationship between the dietary intake and the liver injury during tuberculosis treatment. Second, the adopted FFQ was previously validated for Chinese population. Third, we were able to adjust for common confounding factors for the drug-induced liver injury including age, sex, area, energy intake, diabetes, education level, BMI and outdoor exercise. We also acknowledge a few limitations. First, all participants in this study were Asian, which limited its generalizability. Second, a simplified FFQ was used in this study. We were unable to analyze which specific type of vegetables or cooking oils was associated with the risk of drug-induced liver injury. Third, the sample size was relatively small due to the low incidence of tuberculosis. Fourth, although we used a previously validated FFQ, it is still inherently subject to certain degree of measurement error (22). The measurement error may lead to misclassification in dietary intake, and in turn weaken the association between diet and liver injury.

In conclusion, a high vegetable intake was associated with a low risk of liver injury and liver dysfunction, whereas a low cooking oil intake was associated with a low risk of liver injury and liver dysfunction during tuberculosis treatment. Further studies are required to investigate the mechanism of vegetable and cooking oil consumption in modulating the tuberculosis-drug-induced liver injury.

## Data Availability Statement

The raw data supporting the conclusions of this article will be made available by the authors, without undue reservation.

## Ethics Statement

The studies involving human participants were reviewed and approved by the Ethic Committee of Medicine of Qingdao Center of Disease Control and Prevention (2009-4). The patients/participants provided their written informed consent to participate in this study.

## Author Contributions

AM designed research. LX, CZ, SZ, and YL conducted research. JW and KX analyzed data and wrote the manuscript. AM had primary responsibility for final content. All authors contributed to the article and approved the submitted version.

## Conflict of Interest

The authors declare that the research was conducted in the absence of any commercial or financial relationships that could be construed as a potential conflict of interest.
