# Supplementary material for: Dietary Intake of Vegetables and Cooking Oil Was Associated With Drug-Induced Liver Injury During Tuberculosis Treatment: A Preliminary Cohort Study
Source: Front Nutr. 2021 May 24;8:652311. doi: 10.3389/fnut.2021.652311 (PMC8180911; doi:10.3389/fnut.2021.652311)
Supplement: Supplementary file 1 [file Table_1.docx]

**Supplementary material**

Table S1: The characteristics of the liver injury and dysfunction events.

|  | Liver injury group (n=49) | | Liver dysfunction group (n=141) | |
| --- | --- | --- | --- | --- |
|  | n | Value^1^ | n | Value |
| ALT, U/L^2^ | 49 | 93.0 (110.0) | 141 | 49.0 (31.5) |
| AST, U/L | 49 | 77.0 (89.5) | 141 | 38.0 (34.5) |
| ALB, g/L | 43 | 40.4 (7.9) | 108 | 41.1 (7.7) |

^1^Numerical variables are presented as median (IQR).

^2^ALT, alanine aminotransferase; AST, aspartate aminotransferase; ALB, albumin.
